# Deep-Learning-Based Natural Language Processing of Serial Free-Text Radiological Reports for Predicting Rectal Cancer Patient Survival

**DOI:** 10.3389/fonc.2021.747250

**Published:** 2021-11-17

**Authors:** Sunkyu Kim, Choong-kun Lee, Yonghwa Choi, Eun Sil Baek, Jeong Eun Choi, Joon Seok Lim, Jaewoo Kang, Sang Joon Shin

**Affiliations:** ^1^ Department of Computer Science and Engineering, Korea University, Seoul, South Korea; ^2^ Division of Medical Oncology, Department of Internal Medicine, Yonsei University College of Medicine, Seoul, South Korea; ^3^ Songdang Institute for Cancer Research, Yonsei University College of Medicine, Seoul, South Korea; ^4^ Department of Radiology, Yonsei University College of Medicine, Seoul, South Korea

**Keywords:** rectal cancer, MRI, deep learning, survival prediction, natural language processing (NLP)

## Abstract

Most electronic medical records, such as free-text radiological reports, are unstructured; however, the methodological approaches to analyzing these accumulating unstructured records are limited. This article proposes a deep-transfer-learning-based natural language processing model that analyzes serial magnetic resonance imaging reports of rectal cancer patients and predicts their overall survival. To evaluate the model, a retrospective cohort study of 4,338 rectal cancer patients was conducted. The experimental results revealed that the proposed model utilizing pre-trained clinical linguistic knowledge could predict the overall survival of patients without any structured information and was superior to the carcinoembryonic antigen in predicting survival. The deep-transfer-learning model using free-text radiological reports can predict the survival of patients with rectal cancer, thereby increasing the utility of unstructured medical big data.

## Introduction

The likelihood of cancer patient survival is important information for the patient, their family, and clinicians. Countless studies (1–3) have used serum tumor markers, clinicopathologic features, or clinical trials to predict cancer patient survival, but these methods have exhibited limited effectiveness. Clinicians depend on the clinical histories of patients, their responses to treatment, clinical guidelines, and personal clinical experience. Since the widespread adoption of electronic medical record (EMR) systems (4), medical records have been gradually accumulating in medical institutions (5). The utilization of certain EMR components offers objective and real-time survival information for cancer patients on a precise medical scale.

An obstacle to this objective is that most EMR data are unstructured free-text clinical notes without a standard format. This makes it challenging for the data to have a direct impact on clinical decisions (6–11). Various studies have used unstructured data to assist in clinical decision-making based on deep-learning technology, such as natural language processing (NLP) (12–14). However, the NLP used in previous studies did not include recently introduced state-of-the-art text-comprehension technologies. It thus did not produce key information for clinical decision-making, such as individual patient prognosis prediction. In addition, an advanced analytical method that processes time-series data is required to utilize the EMR data, which accumulate with patient visits.

In this study, we propose a novel deep-transfer-learning model for predicting patient survival based on unstructured free-text data obtained from radiological reports of rectal cancer patients. Radiological reports, which consist of text only and contain a body of findings followed by an impression, provide commonly used types of unstructured EMR data to aid clinical decision-making for cancer patients. Serial radiological reports reflect the changes in the disease status of cancer patients and guide treatment plans. The proposed model obtains the feature vector of the patient from serial radiological reports using a recurrent neural network (RNN) (15) and a state-of-the-art language model pre-trained with public clinical notes. The model predicts the survival risk of the patient from its feature vector using a Cox-proportional hazards (Cox-PH) model. A retrospective study for evaluation revealed that the proposed model successfully predicted rectal cancer patient survival using only free-text radiological reports without any clinical information. Furthermore, we extended the proposed model to a practical algorithm to infer the survival graphs of new patients based on their individual radiological reports.

## Methods

### Study Population and Preprocessing

The retrospective cohort analysis was performed at Yonsei Cancer Center, Seoul, Korea. Based on the EMR review, we identified patients who were diagnosed with rectal cancer between April 2012 and October 2019. The EMR data, including the rectal magnetic resonance imaging (MRI) reports, sexes, ages, and carcinoembryonic antigen (CEA) levels, determined by electrochemiluminescence immunoassay, of the rectal cancer patients, were obtained. A total of 25 radiologists reported MRI reading during that period. Patients without rectal MRI reports were excluded. The study was reviewed and approved by the institutional review and ethics board of the Severance Hospital, Seoul, Korea (IRB no. 4-2020-1003). The requirement of obtaining informed consent was waived owing to the retrospective nature of the study.

### Survival Prediction Model Implementation

**Figure 1:**
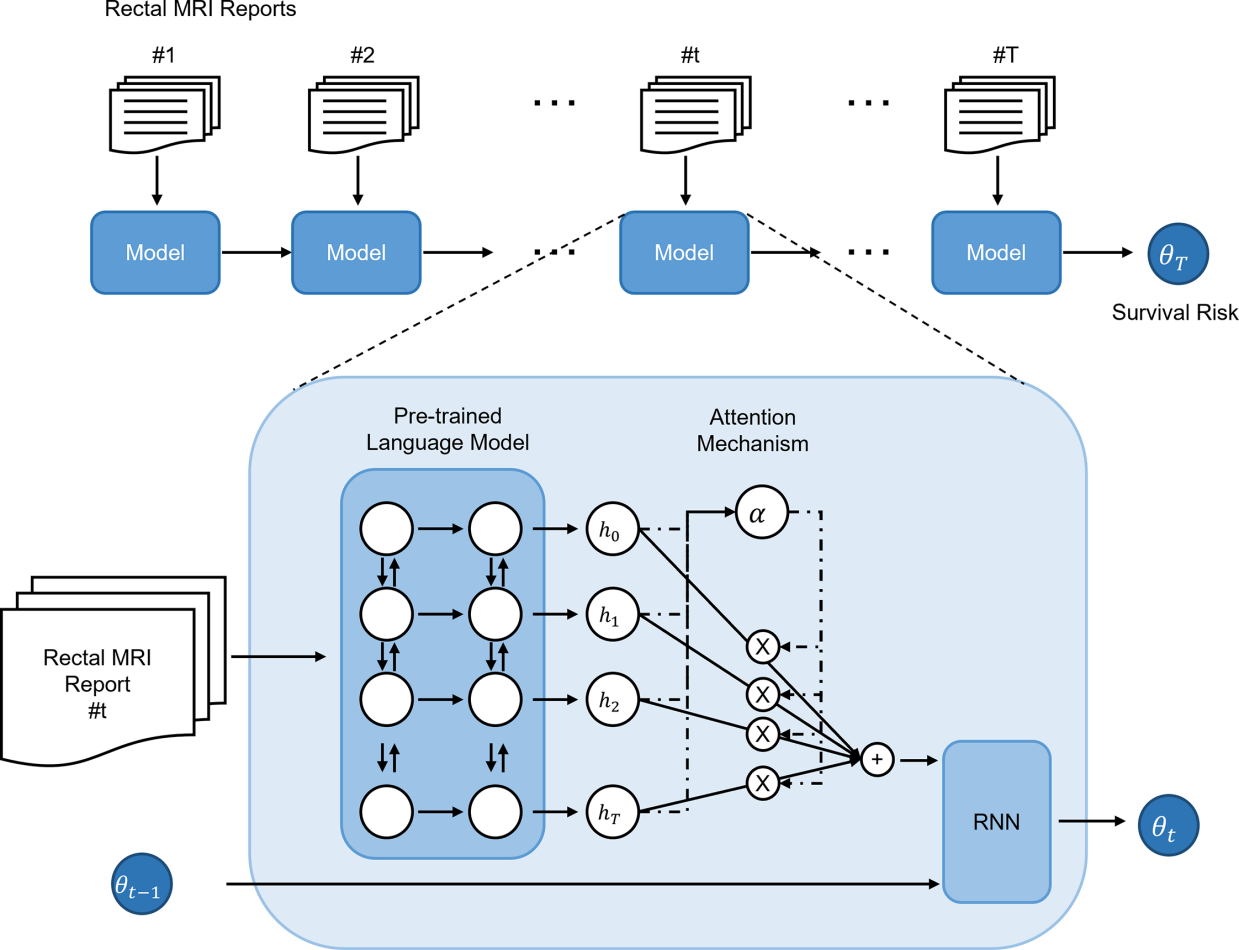
Structure and risk stratification of the deep-transfer-learning model. The radiological report of a patient is first converted into an embedding vector using a pre-trained language model and attention mechanism. If the patient has multiple serial radiological reports, a recurrent neural network (RNN) model aggregates the embedding vectors of the serial reports and generates an embedding vector that represents the patient. Based on the patient embedding vector, a Cox-proportional hazards model predicts the patient’s survival risk.

The primary endpoint of this study was the concordance of the patient risk predicted by the deep-transfer-learning-based survival prediction model and the actual overall patient survival. The proposed model predicted patient survival based on four steps (model structure shown in **Figure 1**). The first step was to convert the input radiological report into an embedding vector that corresponded to each word with a bidirectional encoder representation from transformers (BERT) language model (16), which, among other recent language models, has demonstrated state-of-the-art performance in many fields. The second step was to aggregate the word-embedding vectors into single-feature vectors to represent the radiological reports using an attention technique (17–19) for weighting the more important words in the report. The third step was the serial analysis of multiple radiological reports from a single patient to obtain a patient embedding vector using the gated rectified unit (20) among all RNN implementations. Finally, patient survival was predicted using a Cox-PH (21) model with the patient embedding vector. Detailed descriptions of the loss function and hyperparameters used in the deep-transfer learning are provided in **Supplementary Material**.

### High- and Low-Risk Feature Extraction Through N-Gram Clustering

To extract high- and low-risk features from radiology reports, we obtained N-grams, a continuous sequence of N items in a text or sequence, from the radiology reports for each risk group. Then, N-grams with statistically significant differences in the frequency of appearance for each risk group were selected, and we clustered the selected N-grams using edit distance (22) and determined the representative features from each cluster. See **Supplementary Material** for details.

### Visualization of the Patient Embedding Vectors

To investigate what determines the survival prediction of the deep-transfer learning model, the patient embedding vectors were extracted from the last hidden layer of the model using the first radiological report of the patient. The embedding vectors were then reduced to two-dimensional vectors using the t-stochastic neighbor embedding (t-SNE) algorithm (23) for visualization. The *p-*values were calculated using the log-rank test (24) to compare survival between patient groups stratified by the appearance of the terms.

### N-year Survival Classification

Based on the patient risk, the N-year survival prediction performance was also evaluated using the area under the receiver operating characteristic (AUROC) curve. The patients in the test set were assigned binarized labels with 1-, 2-, 4-, and 5-year survival thresholds. The binarized labels for the patients in the test set were assigned to be positive for patients who survived longer than a certain threshold and negative for patients who did not.

### Statistical Analysis

The performance metrics that were used to evaluate the survival prediction of the deep-transfer-learning model were the C-index (25) and the *p*-value of the log-rank test. To evaluate the association between the patient risk and actual survival time, the Spearman correlation coefficient (*R_s_*) (26) was used. A *p*-value < 0.05 was considered statistically significant. See **Supplementary Material** for details.

## Results

### Patients

In total, the EMR data of 7,402 patients diagnosed with rectal cancer from April 2012 to October 2019 were reviewed, and data of 4,338 patients with available rectal MRI radiological reports were analyzed. Approximately 40% of the patients had two or more serial MRI reports available. The survival cut-off date was October 28, 2019 (median follow-up duration, 49 months). Among the patients, 3,470 were assigned to the training dataset and 868 were assigned to the test dataset (**Supplementary Figure 1**). To maximize the effectiveness of the unstructured data, the clinical data, including the sex, age, and CEA levels, were not used. Only survival or death events were used for the deep-transfer-learning model implementation, which were well balanced between the training and test sets (**Table 1**).

**Table 1:** Patient demographic and clinicopathologic characteristics.

	Training set (n = 3470)	Test set (n = 868)	*p-*value
Sex, No. (%)			
Male	2186 (63.0)	555 (63.9)	0.637
Female	1284 (37.0)	313(36.1)	
Age, No. (%)			
<65	2110 (60.8)	839 (62.2)	0.67
≥65	1360 (39.2)	525 (37.8)	
CEA^a^			
median (range), ng/mL	2.62 (0.0–9561.6)	2.675 (0.0–12382.1)	0.826
>5, No. (%)	911 (26.3)	239 (27.5)	
Survival event, No. (%)			
Alive	2934 (84.6)	715 (82.4)	0.119
Death	536 (15.4)	153 (17.6)	
MRI^b^ reports per patient, No. (%)			
1	2013 (58.0)	503 (57.9)	0.969
≥2	1457 (42.0)	365 (42.1)	

a CEA, carcinoembryonic antigen.

b MRI, magnetic resonance imaging.

### Generation of Survival Prediction of the Deep-Transfer-Learning Model

We developed a novel deep-transfer-learning model to predict patient survival using unstructured serial MRI radiological reports from rectal cancer patients. Firstly, the radiological report of a patient was represented in the form of word-embedded hidden vectors by a pre-trained language model that comprehends the input text as a sequence of words. For the pre-trained language model, we used ClinicalBERT (27), which employs transferred knowledge extracted from public clinical notes. The hidden vectors were then aggregated to construct the feature vector of the patient, which was used as input to the Cox-PH model, which predicts the patient survival risk. For an individual patient with multiple serial rectal MRI radiological reports available, the risk status was predicted by an RNN that combined all the feature vectors obtained from all the available radiological reports.

### Performance of Survival Prediction Deep-Transfer-Learning Model

To evaluate the performance of the deep-transfer-learning model, three comparative experiments were conducted. Firstly, survival predictions obtained from four different language models were compared. An un-pre-trained BERT and three pre-trained BERT language models (original BERT (16), BioBERT (28), and ClinicalBERT (27)) were compared through five-fold cross-validation with the training dataset and external validation using a separate test set (see **Supplementary Material** and **Supplementary Table 1**). The model performance was evaluated using the concordance index (C-index) (25), which is widely used to evaluate the performance of survival prediction models. C-index shows a higher value when the higher survival probability does the model predict for the long-time survived patients. Among the three language models, ClinicalBERT yielded the best survival prediction performance.

Subsequently, we assessed whether survival prediction was improved when multiple serial MRI radiological reports from the patient were utilized. The deep-transfer-learning model predicted the patient survival with serial radiological reports using the RNN, as described earlier. Compared with the single-time-point model (using only the first radiological report from the patient), the serial model (using multiple serial radiological reports from the patient) exhibited an improved C-index for the entire test set (C-index of 0.595 vs. 0.579, p-value < 4.9×10^−2^, **Table 2**). In particular, when two serial radiological reports were used, the survival prediction of the patient was improved in the serial model case (C-index of 0.667) compared with those of patients with single radiological reports (C-index of 0.626). These results imply that the serial model has a better prediction of survival when interpreting subsequent MRI reports that occur following the course of treatment based on the patient’s first MRI report.

**Table 2:** C-index comparison between serial and single time-point models based on visit counts using ClinicalBERT.

Number of serial MRI reports per patient	Patients, No. (%)	Single time-point model	Serial model
1	503 (57.9)	0.555	0.572
2	286 (32.9)	0.626	0.667
3	53 (6.1)	0.486	0.422
4	26 (3.0)	0.638	0.663
≥2	365 (42.1)	0.617	0.638
Total patients	868 (100.0)	0.579	0.595

**Figure 2:**
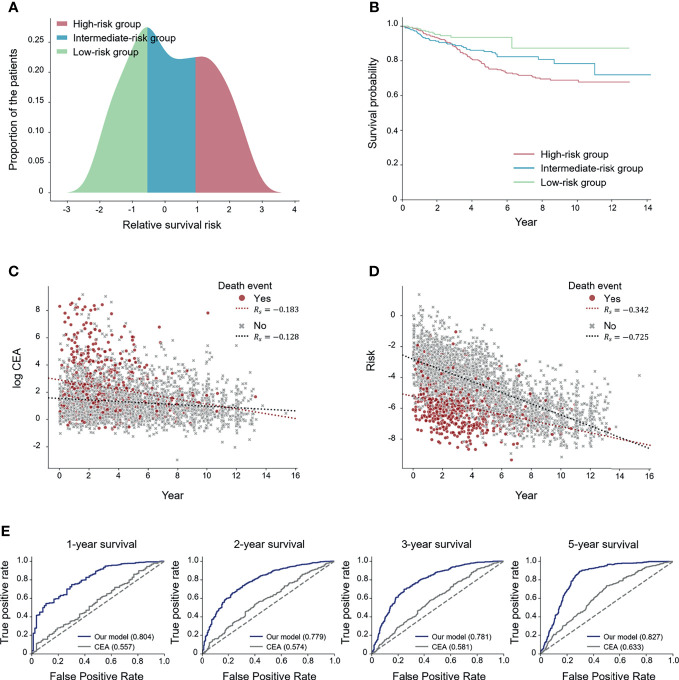
Performance of the deep-transfer-learning-based survival prediction model. **(A)** Distribution of predicted risks of patients in the test set. The patients were divided into high-, medium-, and low-risk groups according to the predicted risks. **(B)** Kaplan–Meier survival graph of each risk group. **(C)** Visualization of the association between log carcinoembryonic antigen (CEA) values and survival times. **(D)** Visualization of the association between the predicted risks and survival times. **(E)** Receiver operating characteristic (ROC) curves for comparing CEA and the predicted risk on N-year survival prediction. The values of each area under the ROC (AUROC) for both the serial model and the CEA are shown (*R_s_*: Spearman’s correlation coefficient).

We divided the patients into three subgroups (high-, intermediate-, and low-risk) based on the predicted risk for each patient. Then, the deep-transfer-learning-based survival prediction model successfully stratified patients into the high-risk group (defined as patients with risks in the top 33rd percentile according to the deep-transfer-learning model) or low-risk group (patients with risks in the bottom 33rd percentile) (log-rank *p*-value < 2.0×10^−3^, **Figures 2A, B**).

In addition, two experiments were conducted to compare the effect of the risk prediction obtained from the tumor marker CEA, which is known to predict the survival outcome of colorectal cancer patients (29), on survival prediction with those of the radiographic reports. Firstly, we visualized how the CEA and risks predicted by our model correlated with the actual survival time using the Spearman correlation coefficient (*R_s_*) (26). The scatter plot results revealed that the predicted risk (*R_s_* = −0.590) had a higher correlation with the actual survival time compared with the CEA (*R_s_* = −0.195) (**Figures 2C, D**). Secondly, we compared the effectiveness of CEA and the risks predicted by our model in forecasting N-year survival using receiver operating characteristic (ROC) curves. Use of the risk obtained through the deep-transfer-learning model yielded higher AUROC values than that of CEA in predicting N-year survival (**Figure 2E**). Additionally, we obtained ROC curves for patients with more than one MRI report (**Supplementary Figure 5**) and found higher AUROC values than the curves for whole patients in most cases. This result means our model performed better when a patient had serial radiological reports.

### Model Interpretation Analysis

**Figure 3:**
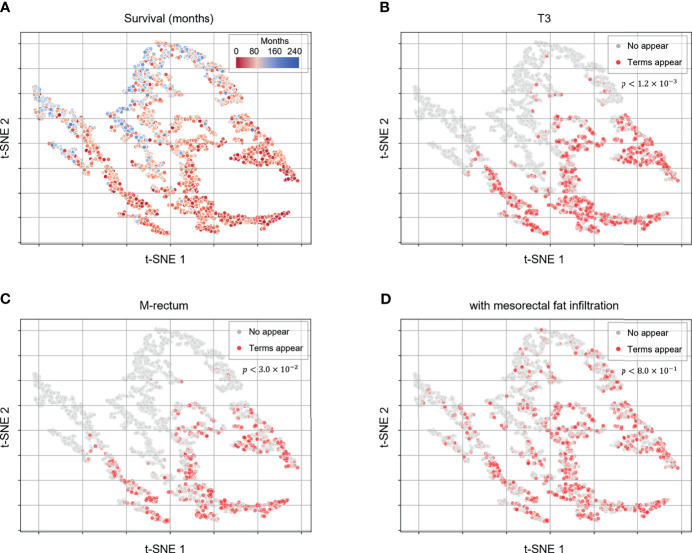
Patient embedding vector scatter plot of the survival and high-risk features. The patient embedding vectors were obtained from the RNN of the deep-transfer-learning model and visualized in a two-dimensional scatter plot using t-distributed stochastic neighbor embedding. **(A)** The survival of each patient is depicted in red (worse survival) or blue (better survival). **(B–D)** The color represents whether clinically meaningful terms (high-risk features: T3, **(B)**; mesorectal fat infiltration, **(C)**; Rb, **(D)** appear (red) in a patient’s radiological report or not (gray). *p*-values were calculated using the log-rank test to compare the survival between patient groups stratified by appearance of the terms.

To obtain deeper clinical insights for utilization of the proposed deep-transfer-learning model, we visualized the embedding vectors of the patients and investigated the hidden knowledge that the deep-transfer-learning model learned from the training data. We considered the hidden vector of the last layer of the model as the patient embedding vector and obtained the scatter plot visualization *via* dimensionality reduction with the t-SNE algorithm (**Figure 3**). When we projected the actual patient survival on the same scatterplot, we identified the distribution of clusters of patients with poor versus favorable survival (**Figure 3A**). To identify which clinicopathologic features were related to high-risk patients, we obtained sequences of words (N-grams) that were prevalent among MRI reports of a high-risk group compared with those of the low-risk group. Then we extracted representative high-risk term features based on clustering the sequence of words. In the same way, the representative low-risk term features were extracted. We found the extracted term features were closely related to the patients’ prognosis and clinically acceptable (**Supplementary Figure 2**). Then we visualized these term features on the scatterplot. Terms that were clinically related to worse prognoses, such as “T3,” “M-rectum,” and “with mesorectal fat infiltration,” were highly prevalent among patients with worse survival [*p*-value < 0.05 between patient groups stratified by the appearance of the terms (**Figures 3B–D**)]. In addition, other terms associated with negative prognoses, such as “enlarged lymph node,” “CRM threatening,” and “suspicious regional lymph node metastases,” were prevalent among patients with worse prognoses. Moreover, a positive prognostic term, “No evidence of significant lymph node enlargements in both pelvic side walls,” was prevalent among patients with favorable survival (**Supplementary Figure 3**).

### New Patient Survival-Graph Generation

Using the trained survival risk prediction model, we produced a survival graph for the radiological reports of a new patient. To obtain reliable survival information, the survival graph of the patient was generated by aggregating the survival information of other patients in the training dataset who were the most similar to the given patient in terms of the predicted risk. After predicting the survival risk value of the new patient using the radiological reports of the patient, the Kaplan–Meier survival curve (30, 31) was plotted using the survival data from 5% of patients in the training dataset with the most similar risk values in the deep-transfer-learning model. **Supplementary Figure 4** presents an example of the survival-graph generation of a new patient. The survival risks predicted by the deep-transfer-learning model for three new rectal cancer patients with MRI radiological reports were plotted in a distribution graph, and Kaplan–Meier survival plots were drawn for each patient.

## Discussion

This study aimed to determine whether the deep-transfer-learning model could predict the survival of cancer patients based only on free-text radiological reports. The results suggest that the deep-transfer-learning model can predict the survival risk of rectal cancer patients by using only rectal MRI reports. Compared with the use of a single-time-point report, survival prediction was improved when multiple serial radiological reports were incorporated by the RNN. The performance improvement of the deep-transfer-learning model when using the past records of the patient can overcome the current problem of non-utilization of accumulated unstructured EMR data because previous approaches require preprocessing steps, which impose an additional workload. Moreover, our proposed method of utilizing serial natural language data for survival prediction is not limited to radiological reports but can be extended to other unstructured data: clinical progress notes and dialogue records between clinicians and patients.

The deep-learning model requires a considerable amount of labeled data (32). However, usually, the amount of training data from a retrospective cohort is limited. Therefore, research using deep-learning models in single institutions has been limited. To overcome the lack of training based on MRI radiological reports, we used transfer learning (33). By comparing three pre-trained language models for transfer learning, we determined that ClinicalBERT, a model trained with clinical notes, was the most effective in predicting cancer patient survival. This result is consistent with the nature of transfer learning; i.e., the transfer is more effective when the source and target tasks are similar (34).

The greatest strength and implication of our deep-transfer model is that it can predict the survival of cancer patients, which is the most important endpoint of any cancer research study. To the best of our knowledge, this is the first study conducted to predict patient survival directly using natural language data. There have been attempts to obtain information about tumor progression automatically based on radiological report text with deep learning (13) but not information about survival itself. Our deep-transfer-learning model directly leverages the patient survival time with the Cox-PH model and predicts patient survival.

When obtaining term features using N-gram extraction and clustering, we systemically identified clinically acceptable high- and low-risk term features, which frequently appeared in the radiological reports of each patient risk group. This analysis can help elucidate clinical features associated with a specific prognosis. Moreover, by including other clinicopathologic factors in the visualization, we expect an in-depth investigation of the association between the clinical features and survival or patient heterogeneity depending on risk. We also developed an algorithm that provides the survival graphs of new patients using their radiological reports based on the trained model and training dataset. Given that the survival graph of a new patient is generated based on the survival information of other patients (with similar response characteristics), we can consider the survival graph reliable. We expect that the survival-graph-generating algorithm can assist clinicians in establishing treatment strategies by providing the corresponding patient survival graphs. The algorithm can be improved by considering other clinical factors as well as radiological reports while collecting survival information from other patients.

Deep-learning techniques intended for image analyses are improving; however, they remain limited to certain modalities (35–38). In this study, radiological reports produced by radiologists were utilized to train the deep-transfer-learning model. Hence, the training included not only anatomical findings from the imaging scans but also clinical insights that reflected the experiences of expert radiologists, which further highlights the strength of this study. The increase in data required for medical decision-making and the rapid proliferation of medical data obscure the decision-making of clinicians (39, 40). In this context, our deep-transfer-learning model, which provides survival risk prediction using a free-text report, will not only help reduce clinical errors but also enhance clinical decision-making. It can thus serve as a foundation for an artificial-intelligence-based clinical decision supporting system utilizing medical big data.

This study has inherent limitations and biases: it was a retrospective study conducted at a single institution. It is not expected that a deep-transfer-learning model trained in one institution will work properly at other institutions. However, we did not include any institution-specific preprocessing or labeling during training. In fact, although MRI reports by 25 radiologists with their own styles were used, every radiologist followed the consensus guideline for rectal MRI reading and therefore all the essential information were included (41). Moreover, our deep learning model succeeded in predicting survival despite having trained based on various styles of reports, which shows the robustness of our model. Based on these results, when our model is adapted to other institutions, we expect that the model algorithm will work properly after retraining with the accumulated natural language data of those institutions (free-text radiological reports).

The main objective of our study is to demonstrate that it is possible to utilize the accumulated unstructured medical text data for a clinical purpose. It is regarded that five variables for a decision-making are the limit of human cognitive capacity (40), however, the recent increase of biomarkers and therapeutical options may threaten to overwhelm a clinician’s cognitive capacity. Therefore, a machine that reads radiological reports in advance and provides an organized information related to survival can be an effective way to reduce clinical errors, even though clinicians are already proficient enough.

We trained our deep-transfer-learning model only with rectal MRI reports from rectal cancer patients. Rectal MRI has been regarded as a gold standard modality for rectal cancer staging and treatment planning, and relevant anatomic landmarks or key features that should be addressed in the MRI reports have been validated (42). Thus, rectal MRI reports are sound unstructured EMR data candidates for training by deep transfer learning to predict patient survival. However, our deep-transfer-learning model structure does not contain any rectal cancer or rectal MRI-dependent features. In fact, our model *per se* is a deep-transfer-learning model that uses a serial natural language model to predict survival, and thus our model has tremendous application potential that can be extended to the use of any type of unstructured EMR data from any type of cancer patient.

## Data Availability Statement

The implementation code of this study is available at https://github.com/SunkyuKim/rectal_mri_survival. The original contributions presented in the study are included in the article/**Supplementary Material**, further inquiries can be directed to the corresponding authors.

## Ethics Statement

The study was reviewed and approved by the institutional review and ethics board of the Severance Hospital, Seoul, Korea (IRB no. 4-2020-1003). The requirement for informed consent was waived owing to the retrospective nature of the study.

## Author Contributions

SK, C-kL, JK, and SS contributed to the concept and design of the study. All authors contributed to the acquisition, analysis, or interpretation of data. SK, C-kL, JK, and SS drafted the manuscript. SK, C-kL, JK, and SS contributed to the critical revision of the manuscript for important intellectual content. SK, C-kL, YC, EB, and JC contributed to the statistical analysis. SK, C-kL, YC, JL, JK, and SS provided administrative, technical, or material support. SK, C-kL, JK, and SS supervised the study. All authors contributed to review and critical revision of the manuscript and approved the final version of the manuscript.

## Funding

This study was supported by a Severance Hospital Research fund for Clinical excellence (SHRC) (C-2020-0030), the Big Data Center at the National Cancer Center of Korea (2020-datawe08), the Ministry of Health & Welfare, Republic of Korea (grant number: HR20C0021(3)), and Korea University Grant.

## Conflict of Interest

The authors declare that the research was conducted in the absence of any commercial or financial relationships that could be construed as a potential conflict of interest.

## Publisher’s Note

All claims expressed in this article are solely those of the authors and do not necessarily represent those of their affiliated organizations, or those of the publisher, the editors and the reviewers. Any product that may be evaluated in this article, or claim that may be made by its manufacturer, is not guaranteed or endorsed by the publisher.
