# Supplementary material for: Deep-Learning-Based Natural Language Processing of Serial Free-Text Radiological Reports for Predicting Rectal Cancer Patient Survival
Source: Front Oncol. 2021 Nov 17;11:747250. doi: 10.3389/fonc.2021.747250 (PMC8635726; doi:10.3389/fonc.2021.747250)
Supplement: Supplementary file 1 [file DataSheet_1.docx]

Supplementary Material

# Supplementary Methods

## *Deep-transfer-learning-based survival prediction model*

Using a pre-trained language model is a key technique in natural language processing. A language model learns text features, including the meaning of words and grammar, from a tremendous amount of text in an unsupervised manner. After pre-training the language model, we can obtain word embedding vectors and transfer them to another text-comprehension model as input data.

Recently, contextualized language models have been introduced and have demonstrated the best performance in text-comprehension tasks. The contextualized word embedding model provides different embedding vectors for the same word depending on the context of the sentence in which that word appears, whereas the traditional word embedding model provides the same embedding vector for the same word.

In this study, we used a state-of-the-art language model, namely, the bidirectional encoder representations from transformers (BERT) (1). BERT is a deep-learning-based language model composed of a bidirectional transformer network. BERT is pre-trained with a large amount of text corpora, including Wikipedia and BookCorpus. Owing to the success of BERT, extensions of BERT, such as BioBERT (2) and ClinicalBERT (3), have been introduced. BioBERT is an extension of the BERT model and is pre-trained with an entire corpus of PubMed abstract texts based on the original pre-trained BERT model. ClinicalBERT is likewise an extension of the BERT model and is pre-trained with a large clinical record dataset, MIMIC-3 (4) based on the original pre-trained BERT model. We expected that the pre-trained model would help our survival prediction model comprehend clinical terms as well as the general natural language. The word embedding vectors were aggregated into a single vector that represented the radiological reports. The deep-transfer-learning model used a self-attention mechanism to create feature vectors for the radiological reports. During the construction of the vector of the radiological report, the attention mechanism (5-7) measured the importance of each word according to its importance in the report. Based on the importance values, the word embedding vectors were aggregated to generate a report-embedding vector.

The patient's multiple radiological reports were then converted into a patient embedding vector that represented the patient’s serial radiological reports. To analyze multiple reports serially, the gated rectified unit (GRU) (8), which is an implementation of the recurrent neural network (RNN) (9), was used. GRU and long-short term memory (LSTM) (10) are improved versions of the RNN and are used to address the vanishing gradient problem. GRU is similar to LSTM but has fewer trainable parameters. Moreover, GRU is known to perform better on smaller datasets than LSTM.

Finally, the patient's survival risk was predicted using a Cox-proportional hazards (Cox-PH) (11) model and the patient embedding vectors. The Cox-PH model was trained to predict the patient’s risk in concordance with the order of actual survivals.

Five-fold cross-validation was performed to obtain optimal hyperparameters using the training dataset in comparing the three pre-trained language models. The optimal hyperparameters used in the final model had 200 dimensions for the attention layer and 768 dimensions for word embedding and the other hidden layers. Tanh (12) was used for the activation function. The parameters were optimized using the Adam optimizer with a learning rate of 1×10^−4^ and L2 regularization of 3×10^−7^. For model implementation, the Pytorch framework (13) and Python-based deep learning library were used.

## *Cox-PH model and statistical analysis*

We combined the pre-trained language model with the Cox-PH model. The Cox-PH model predicts the cancer patient hazard ratio after considering censored patient samples. The hazard ratio is a measure of the likelihood that the patient will die. A lower hazard ratio means the patient is more likely to survive. The Cox-PH model is defined as follows.

$h\left( t | x_{i} \right)=h_{0}\left( t \right)\exp\phi_{i}$ (1)

$\phi_{i}=x_{i}^{T}\theta$ (2)

Here, $\phi_{i}$ is the log-hazard ratio for patient $i$, and $\theta$ is the set of trainable parameters of the model. The objective function of the Cox-PH model is a negative partial log-likelihood, defined as follows.

$\hat{\theta} =argmin_{\theta}-\sum_{i:O\left( i \right)=1} \left( \phi_{i}-\log\sum_{j:t_{j}\geq t_{i}} \phi_{j} \right)$, (3)

where *t* is the patient’s survival time. The condition $t_{j}\geq t_{i}$ refers to the selection of samples whose survival time is longer than the $i$th patient’s survival time. $O\left( i \right)=1$ is an indicator that has a value of one when the death event of the $i$th patient sample is observed.

The concordance index (C-index) is a rank correlation between the predicted risk and real survival considering censored survival data. The C-index is defined as follows.

$c=\frac{1}{N}\sum_{i:O\left( i \right)=1} \sum_{j:t_{j}\geq t_{i}} I[\theta_{i}>\theta_{j}]$, (4)

where the notations are the same as those explained above. $I[\theta_{i}>\theta_{j}]$ refers to an indicator function that is equal to unity when the *i*th patient’s predicted risk $\theta_{i}$ is larger than the *j*th patient’s predicted risk $\theta_{j}$.

The log-rank test (14) is a statistical hypothesis test for the comparison of the distribution of survival of the two data. It is extensively used in clinical trials to demonstrate that there is a difference in survival between the two groups by using the time to the event and the censoring information. The log-rank statistic is defined as follows.

$Z=\frac{\sum_{j=1}^{J} \left( O_{i,j}-E_{i,j} \right)}{\sum_{j=1}^{J} V_{i,j}}\underset{\to}{d}\mathcal{N}\left( 0,1 \right)$ (5)

$E_{i,j}=N_{i,j}\frac{O_{j}}{N_{j}} , V_{i,j}=E_{i,j}\left( \frac{\begin{matrix} N_{j} & O_{j} \end{matrix}}{N_{j}} \right)\left( \frac{\begin{matrix} N_{j} & N_{i,j} \end{matrix}}{N_{j}-1} \right)$ (6)

Here, $N_{i,j}$ denotes the number of subjects “at risk” (who have not yet had an event or been censored) since time *j* in group *i.* $O_{j}$ refers to the observed number of events in the groups at time *j*.

Spearman's rank correlation coefficient (15) is a statistical method for assessing how well two variables can explain each other. Spearman’s correlation between the two variables is equal to the Pearson correlation between the rank values of the two variables. The Spearman correlation, $R_{s}$, is defined as follows.

$R_{s}=\rho_{rg_{X},rg_{Y}}=\frac{cov\left( rg_{X},rg_{Y} \right)}{\sigma_{rg_{X}}\sigma_{rg_{Y}}},$ (7)

where *r_g_* denotes ranks, $\rho$ denotes the Pearson correlation coefficient, *cov* indicates the covariance, and $\sigma_{rg}$ indicates the standard deviation of the rank variables.

## *Term feature extraction through N-gram clustering*

To extract term features specific to each risk group, we first extracted N-grams (16) from the radiology reports for each risk group. An N-gram is a continuous sequence of N items in a text or sequence. In a general case of text data, items can be syllables, letters, or words. In this study, the item refers to the token obtained with the WordPiece tokenizer (17) provided by BERT, and N was set to 10 to extract term features of sufficient length. For N-grams extracted from each risk group, Fisher's exact test was used to determine whether there were statistically significant differences in the frequency of appearance.

When we obtained significant N-grams from each risk group in this way, we saw that very similar N-grams were included as independent individuals. This noise occurs because adjacent N-grams were drawn together in the process of extracting all possible N-grams from given texts, which was an essential limitation of N-gram analysis. To obtain clinically meaningful text features, representative forms must be extracted from N-grams of similar appearance. To address the issue, the similarity between N-grams was measured using the edit distance (18) used to measure the similarity between text or gene sequences. Hierarchical clustering was performed based on the obtained similarity, and final text features were extracted based on the N-grams contained in each cluster.

# References for Supplementary Methods

1. Devlin J, Chang M-W, Lee K, Toutanova K. Bert: Pre-training of deep bidirectional transformers for language understanding. arXiv preprint arXiv:181004805. 2018.

2. Lee J, Yoon W, Kim S, Kim D, Kim S, So CH, et al. BioBERT: a pre-trained biomedical language representation model for biomedical text mining. Bioinformatics. 2020;36(4):1234-40.

3. Huang K, Altosaar J, Ranganath R. Clinicalbert: Modeling clinical notes and predicting hospital readmission. arXiv preprint arXiv:190405342. 2019.

4. Johnson AE, Pollard TJ, Shen L, Lehman LW, Feng M, Ghassemi M, et al. MIMIC-III, a freely accessible critical care database. Sci Data. 2016;3:160035.

5. Bahdanau D, Cho K, Bengio Y. Neural machine translation by jointly learning to align and translate. arXiv preprint arXiv:14090473. 2014.

6. Vaswani A, Shazeer N, Parmar N, Uszkoreit J, Jones L, Gomez AN, et al., editors. Attention is all you need. Advances in neural information processing systems; 2017.

7. Lin Z, Feng M, Santos CNd, Yu M, Xiang B, Zhou B, et al. A structured self-attentive sentence embedding. arXiv preprint arXiv:170303130. 2017.

8. Cho K, Van Merriënboer B, Gulcehre C, Bahdanau D, Bougares F, Schwenk H, et al. Learning phrase representations using RNN encoder-decoder for statistical machine translation. arXiv preprint arXiv:14061078. 2014.

9. Rumelhart DE, Hinton GE, Williams RJ. Learning Representations by Back-Propagating Errors. Nature. 1986;323(6088):533-6.

10. Hochreiter S, Schmidhuber J. Long short-term memory. Neural computation. 1997;9(8):1735-80.

11. Cox DR. Regression models and life‐tables. Journal of the Royal Statistical Society: Series B (Methodological). 1972;34(2):187-202.

12. Fan E. Extended tanh-function method and its applications to nonlinear equations. Physics Letters A. 2000;277(4-5):212-8.

13. Paszke A, Gross S, Massa F, Lerer A, Bradbury J, Chanan G, et al., editors. Pytorch: An imperative style, high-performance deep learning library. Advances in neural information processing systems; 2019.

14. Mantel N. Evaluation of survival data and two new rank order statistics arising in its consideration. Cancer Chemother Rep. 1966;50(3):163-70.

15. Myers JL, Well A, Lorch RF. Research design and statistical analysis: Routledge; 2010.

16. Broder AZ, Glassman SC, Manasse MS, Zweig G. Syntactic clustering of the Web. Comput Networks Isdn. 1997;29(8-13):1157-66.

17. Sennrich R, Haddow B, Birch A. Neural machine translation of rare words with subword units. arXiv preprint arXiv:150807909. 2015.

18. Ristad ES, Yianilos PN. Learning string-edit distance. Ieee T Pattern Anal. 1998;20(5):522-32.

# Supplementary Figures

#
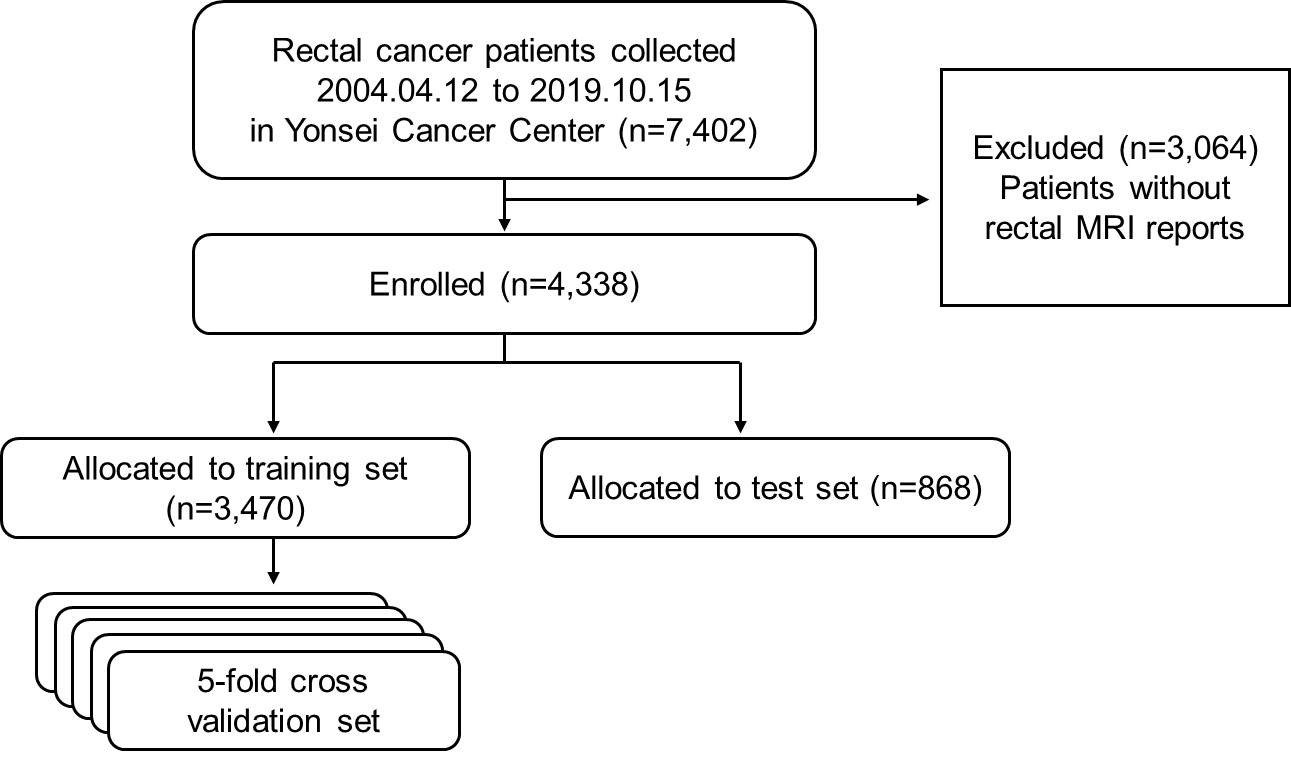


**Supplementary Figure 1. Study flow chart.** In this study, among 7,402 rectal cancer patients with EMR data from April 2012 to October 2019 in Yonsei Cancer Center, 4,338 patients with MRI radiological reports were analyzed; 3,470 patients were assigned to the training dataset and 868 were assigned to the test dataset.


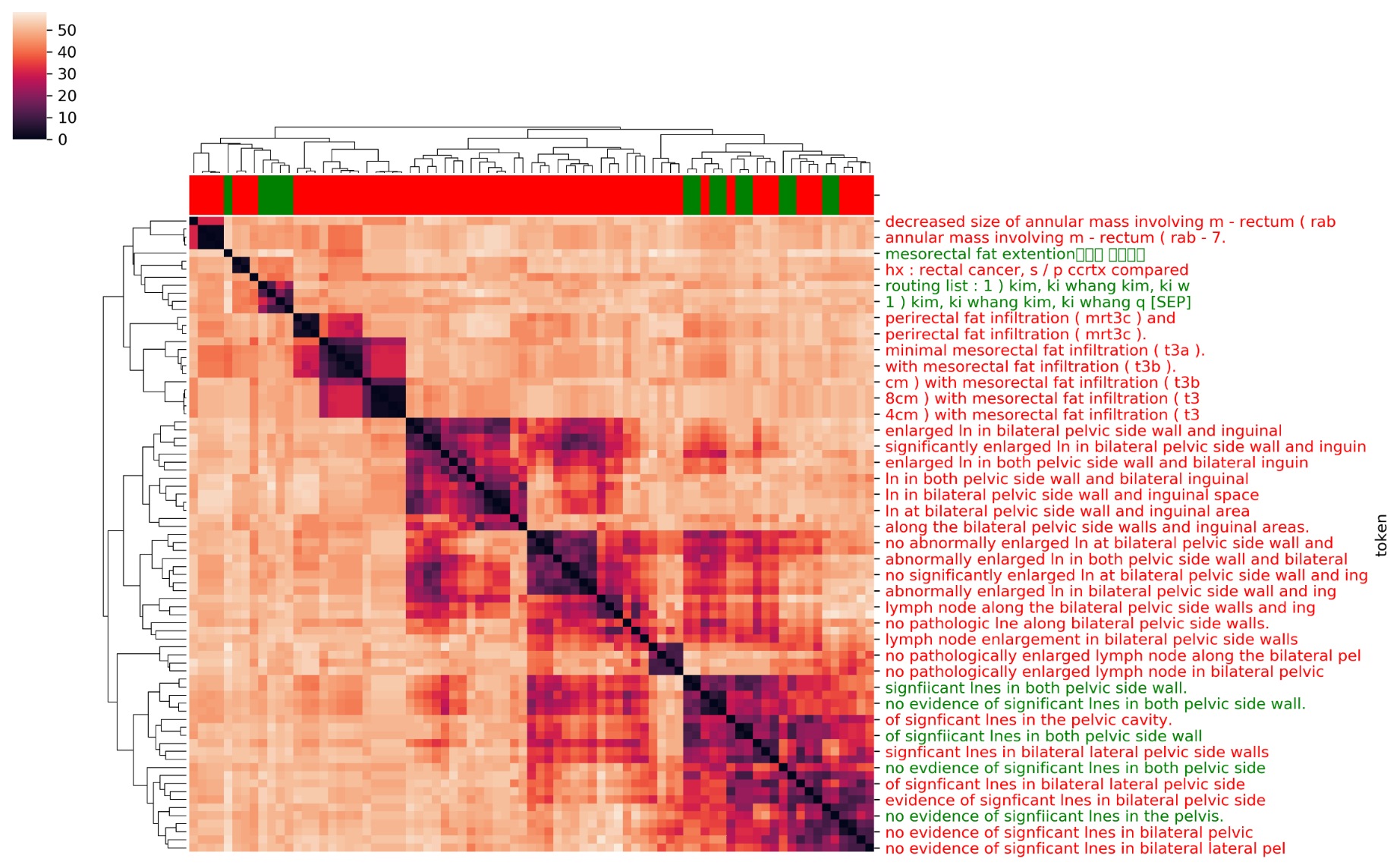


**Supplementary Figure 2. A cluster map of textual features that specifically appear in the radiology reports of each risk group.** After extracting N-grams from the radiology report for each risk group, Fisher's exact test was used to select N-grams with statistically significant differences in frequency of appearance. Then we calculated the edit distance by calculating the text distance between N-grams to get the cluster map. Representative textual features for each risk group can be extracted from each cluster.


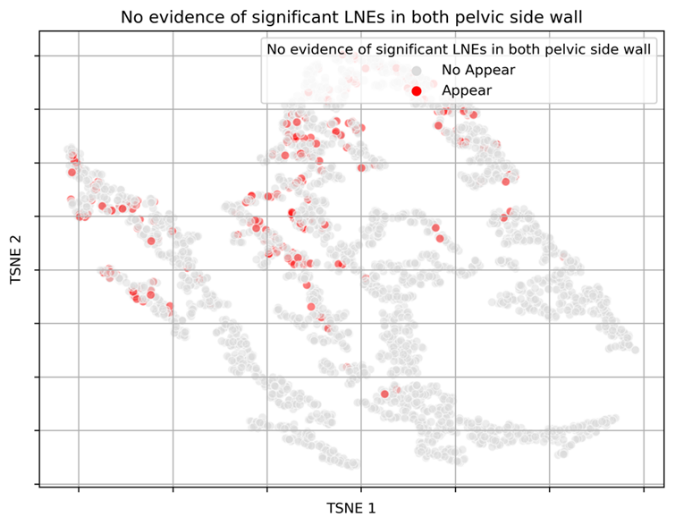

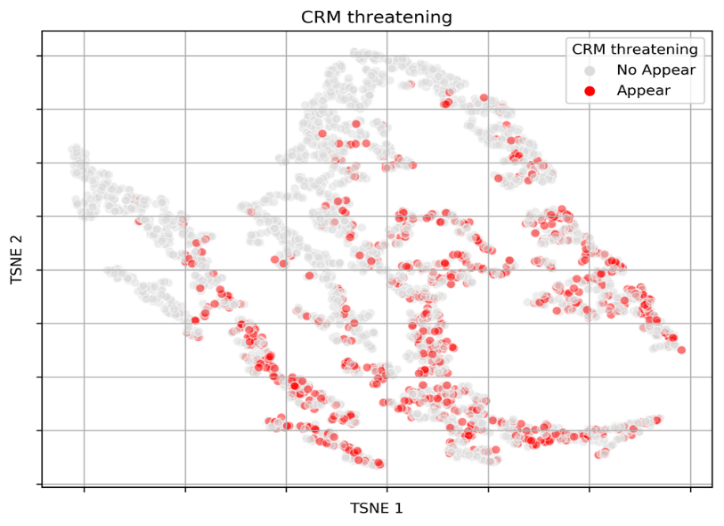

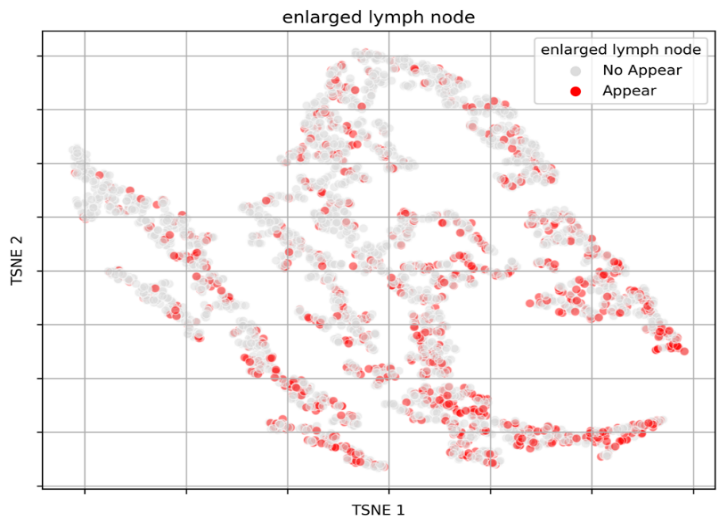


**A**

**B**


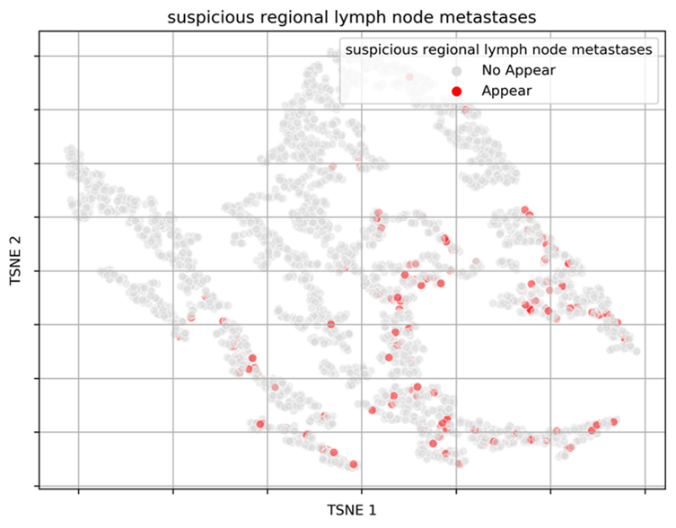


**C**

**D**

**Supplementary Figure 3. Visualization of patient embedding vectors using t-distributed stochastic neighbor embedding (t-SNE) in terms of survival and clinically meaningful features.** The patient embedding vectors were obtained from recurrent neural network (RNN) of the deep-transfer-learning model and visualized in 2-dimension using t-SNE. The color represents whether clinically meaningful terms (enlarged lymph node, A; CRM threatening, B; suspicious regional lymph node metastases, C; no evidence of significant lymph node enlargements (LNEs) in both pelvic side walls, D) appears (red) in a patient’s radiology report or not (gray).

#
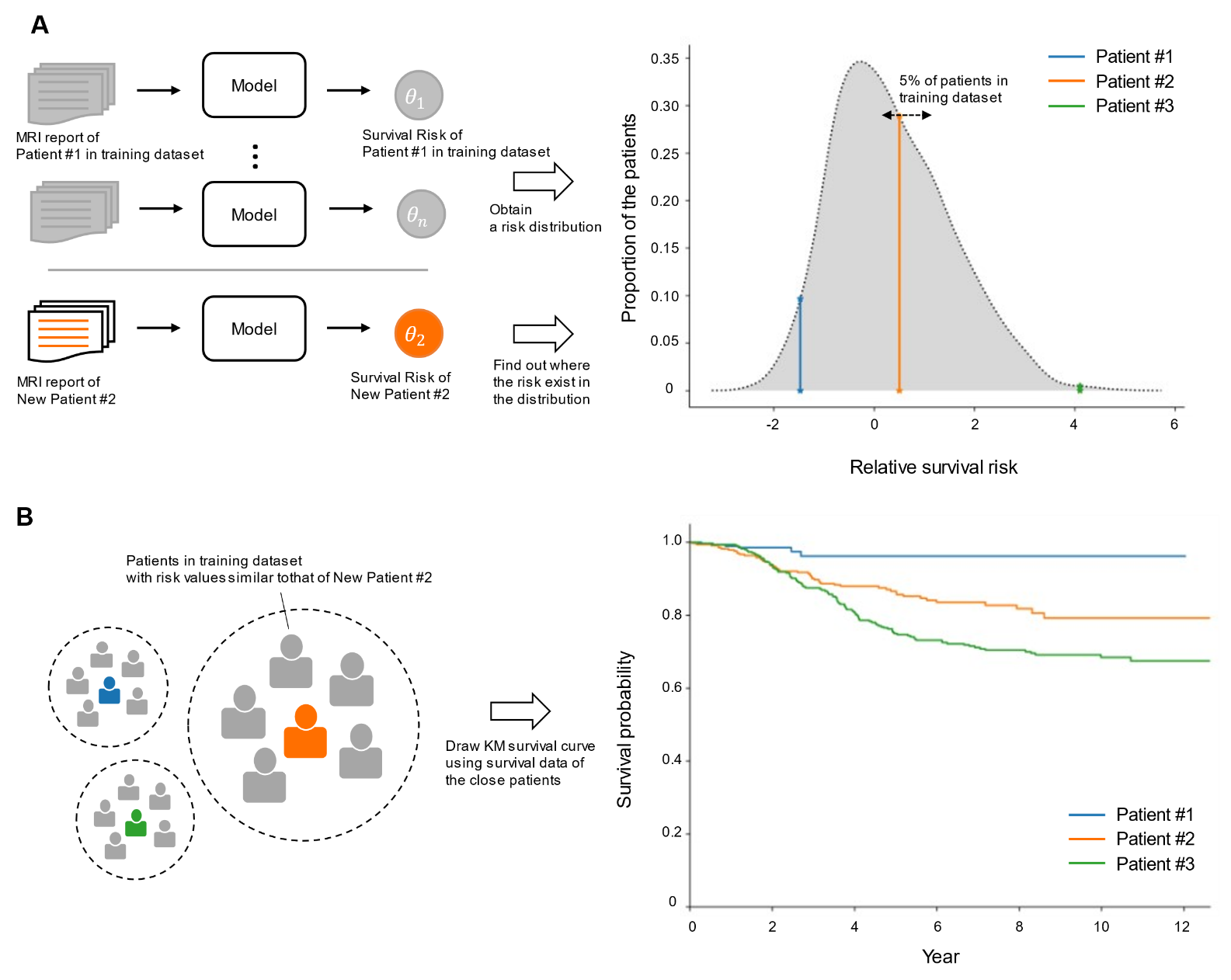


**Supplementary Figure 4. Generation of survival graph of new patients.** (A) Based on trained survival risk prediction model, the survival risks distribution was obtained using the MRI reports of patients in training dataset. MRI report of a new patient is then used as an input of the prediction model and the new patient’s survival risk is calculated. In the distribution plot (right), the dotted line means the distribution of predicted risk values of training dataset patients, and the colored lines mean the predicted risk values of new patients’ MRI reports. (B) Kaplan–Meier survival curve of each new patient was plotted using the survival data from 5% of patients in the training dataset with the most similar risk values to that of the new patient.


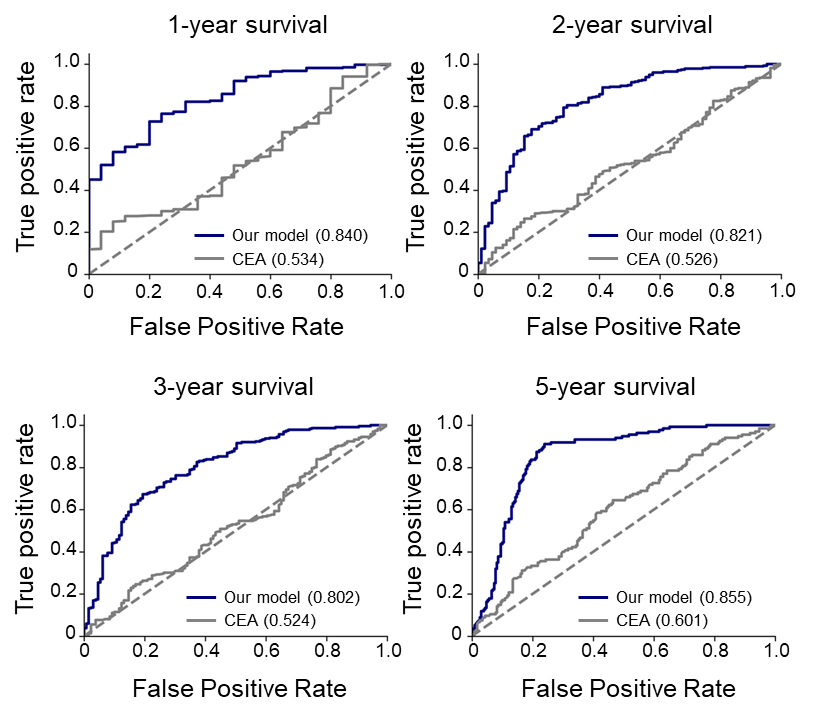


**Supplementary Figure 5. Receiver operating characteristic (ROC) curves for comparing CEA and the predicted risk on N-year survival prediction for patients having multiple MRI reports (n=365).** Receiver operating characteristic (ROC) curves for comparing CEA and the predicted risk on N-year survival prediction for patients having two or more MRI reports. The values of each area under the ROC (AUROC) for both the serial model and the CEA are shown (𝑅_𝑠_: Spearman’s correlation coefficient).

| **Supplementary Table 1. C-index comparison serial models with three pre-trained language models using five-fold cross-validation and external test set** | | | | | | |
| --- | --- | --- | --- | --- | --- | --- |
|  | **CV 1** | **CV 2** | **CV 3** | **CV 4** | **CV 5** | **Test** |
| *Un-pre-trained BERT* | *0.581* | *0.607* | *0.579* | *0.590* | *0.598* | *0.547* |
| OriginalBERT | 0.603 | **0.608** | 0.557 | **0.607** | 0.61 | 0.58 |
| BioBERT | 0.575 | 0.567 | 0.58 | 0.561 | 0.598 | 0.584 |
| ClinicalBERT | **0.618** | 0.569 | **0.581** | 0.582 | **0.618** | **0.595** |
| CV, Cross-validation; BERT, Bidirectional Encoder Representations from Transformers | | | | | | |
